# Walking and Hippocampal Formation Volume Changes: A Systematic Review

**DOI:** 10.3390/brainsci15010052

**Published:** 2025-01-08

**Authors:** Mohamed Hesham Khalil

**Affiliations:** Department of Architecture, University of Cambridge, Cambridge CB2 1PX, UK; mhmhk2@cam.ac.uk

**Keywords:** walking, hippocampus, hippocampal volume, plasticity, subiculum, parahippocampal gyrus, step count, physical activity, navigational training, green space

## Abstract

Background/Objectives: Sustaining the human brain’s hippocampus from atrophy throughout ageing is critical. Exercise is proven to be effective in promoting adaptive hippocampal plasticity, and the hippocampus has a bidirectional relationship with the physical environment. Therefore, this systematic review explores the effects of walking, a simple physical activity in the environment, on hippocampal formation volume changes for lifelong brain and cognitive health. Method: PubMed, Scopus, and Web of Science were searched for studies on humans published up to November 2022 examining hippocampal volume changes and walking. Twelve studies met the inclusion criteria. Study quality was assessed using the PEDro scale and ROBINS-I tool. A narrative synthesis explored walking factors associated with total, subregional, and hemisphere-specific hippocampal volume changes. Results: Overall, walking had positive effects on hippocampal volumes. Several studies found benefits of higher-intensity and greater amounts of walking for total hippocampal volume. The subiculum increased after low-intensity walking and nature exposure, while the parahippocampal gyrus benefited from vigorous intensity. The right hippocampus increased with spatial navigation during walking. No studies examined the effect of walking on the dentate gyrus. Conclusions: This systematic review highlights walking as a multifaceted variable that can lead to manifold adaptive hippocampal volume changes. These findings support the promotion of walking as a simple, effective strategy to enhance brain health and prevent cognitive decline, suggesting the design of physical environments with natural and biophilic characteristics and layouts with greater walkability and cognitive stimulation. Future research is encouraged to explore the hippocampal subregional changes instead of focusing on total hippocampal volume, since the hippocampal formation is multicompartmental and subfields respond differently to different walking-related variables.

## 1. Introduction

The hippocampus is one of the human brain’s most complex and challenging neuroplastic structures. Each of the right and left-hippocampal structures consists of the dentate gyrus, Cornu Ammonis (CA1–3) regions, subiculum, and parahippocampal gyrus [1], forming an intricate neural circuit that is sophisticatedly connected to other brain regions such as the cortex, amygdala, striatum, and thalamus. The right hippocampus plays a more prominent role in spatial memory and navigation, while the left supports verbal memory to a greater degree in humans [2]. The hippocampus witnesses atrophy throughout ageing, but its structure has been shown to benefit from exercise, and the hippocampus has a bidirectional relationship with its environment. Thus, walking can be a simple habit with long-term benefits on brain health and preventing cognitive decline.

Exercise has a primary effect on the dentate gyrus cerebral blood volume [3], but whether it impacts adult neurogenesis in humans is debatable. The hippocampal dentate gyrus is associated with the current debate on adult hippocampal neurogenesis, a process through which new neurons are born in the dentate gyrus. Although widespread in non-mammalian vertebrates, neurogenesis is reduced in mammals [4]. Since the groundbreaking discovery that newborn neurons are generated in the adult rat brain [5], and after the incorporation of modern labelling techniques such as bromodeoxyuridine (BrdU) [6], a growing body of the literature, including rodent studies, has recently been considered as giving hope for the human brain [7,8]. However, human research has been less conclusive since the first suggestion of neurogenesis in the adult human brain [9], using postmortem brain samples with BrdU for diagnostic purposes. On the one hand, some researchers have quantified various processes in the dentate gyrus [10,11,12,13,14], showing that the brain region is active. Interestingly, those processes persist even into the tenth decade of life. On the other hand, several studies support skepticism about adult neurogenesis. Cipriani et al. [15] found that neurogenesis declines sharply after childhood, with few DCX+ cells in adults, concluding that it is negligible in adulthood. Sorrells et al. [16] confirmed this, showing a lack of progenitor cells in the subgranular zone during development and no young neurons in adults aged 18–77. Duque and Spector [17] warned that neural stem cell interventions may disrupt brain homeostasis, advocating for neuron preservation instead. Snyder [18] proposed reevaluating neurogenesis’s role in cognition, while Simard et al. [19] called for diverse methods and broader definitions to advance the understanding of neurogenesis. Despite the debate, this systematic review hypothesizes that walking can be a method to sustain neurogenesis.

Intense physical activity may enhance the hippocampal CA2/CA3 volume in young adults [20]. The hippocampus CA regions are integral to spatial navigation. Place cells, which are mostly studied on rodent subjects, are neurons that fire when the subject is in a specific location in their environment and are prominently found in the CA regions. CA3, in particular, generates spatial representations and completes patterns of spatial information, while CA1 refines these representations by integrating them with temporal contexts [21]. This systematic review can face two significant challenges in CA: methodological limitations and high-intensity activity can inhibit proper observation between walking and CA volume changes or environment-related cognitive mapping.

The hippocampal subfield subiculum is less studied, but an increase in subiculum volume is associated with gains in spatial memory [22]. What distinguishes the subiculum is that it plays an important role in preventing the hypothalamic–pituitary–adrenal (HPA) axis in response to stress [23]. Physical activity decreases cortisol levels [24], cortisol is used as a biomarker to indicate dysregulation of the HPA axis, and meditation reverses the effects of stress [25]. Thus, the relationship between walking, the environment, and the subiculum is worth exploring.

Last but not least, exercise appears to have extensive effects on parahippocampal function, as demonstrated through 20 human studies and 8 animal model studies. Exercise increases neural excitability, white matter and grey matter, cerebral blood flow, markers of synaptic plasticity, and more in the parahippocampal gyrus [26].

Changes in hippocampal volume are sensitive to the respective hemisphere location and subfields. Volume changes, if any, can reflect various processes, including synaptic remodeling and dendritic branching [27,28]. Those plasticities can take place at both the right and left hippocampus that mark hemispheric differences [29]. The right hippocampus predominantly engages in spatial memory processing and shows greater volume changes in response to spatial navigation tasks, while the left hippocampus, specializing in verbal memory and narrative processing, exhibits distinct plasticity patterns during language acquisition and semantic learning [2]. Therefore, interpretation of any increase in hippocampal volume should be sensitive to the hemisphere and subregion, if specified.

The complexity of the hippocampus, particularly in the human brain, leads to four major challenges unique to our human species. First, humans, unlike other species, have less or arguably negligible adult hippocampal neurogenesis in their dentate gyrus compared to other species, such as rodents, leading to the question of how to at least sustain the adult neurogenesis rate that persists until the tenth decade of life, possibly through exercise that may prevent hippocampal atrophy. Second, humans have free-will to exert a motor activity, even such as walking, in a non-structured way that is often absent, leading to the question of what are the minimum walking parameters needed for it to function as a form of exercise that is proven to be effective [30,31], which may help prevent hippocampal atrophy or increase its volume, not only in the dentate gyrus but also beyond this subfield. Third, due to the structural complexity of the hippocampus, methodological limitations in humans often inhibit the parameter–subfield relationship, leading to the question of the sensitivity of each hippocampal subregion to certain variables. Last but not least, the effect of the environment cannot be separable from the effect of walking itself on hippocampal plasticity, which is another important variable to be taken into account when analyzing the effect of walking on hippocampal structural plasticity changes. Hence, this systematic review aims to explore the relationship between walking and hippocampal volume changes to explore the environmental affordance parameters (respective to walking itself and the environment) associated with volume changes in the hippocampal formation.

This systematic review addresses an important gap in the literature by exploring how walking affects the size of different parts of the hippocampus. It specifically looks at how both walking-related factors and walking-associated environmental conditions might influence changes in the hippocampus and hippocampal subregion volume. Earlier, a systematic review and meta-analysis was published a few years back showing that aerobic exercise had significant effects on certain hippocampal regions in comparison to control conditions, suggesting that aerobic exercise can be useful for preventing age-related hippocampal deterioration [31]. Another systematic review and meta-analysis showed that among adults, moderate-intensity continuous training and resistance training could potentially augment the hippocampus volume [30]. Thus, an up-to-date systematic review with a focus only on walking that is partially a form of physical activity could provide very useful insights for research on hippocampal neuroplasticity and cognitive function.

The contribution of this systematic review can also provide an additional lens of insights for recent reviews on sedentary behaviors and brain health [32] and on sedentary behaviors and BDNF [33]. Walking is suggested to be a promising free-living physical activity, where free-living physical activities can similarly be quantified as MET minutes per week [34], which aligns with the recent novel framework of environmental affordance for physical activity [35]. Last but not least, this systematic review aims to explore how walking can increase the hippocampal volume, if possible, since the latter is inversely associated with the rate of depression [36,37,38], and how it can improve cognition and mental health by spending time outside the house [39,40]. This systematic review also contributes towards enriching the novel theory of environmental affordance for physical activity [35], which considers walking as one of the ways through which the environment can promote physical activity.

## 2. Method

### 2.1. Research Strategy

The literature search was carried out using three databases: PubMed, Web of Science, and Scopus. The findings are presented following the guidelines outlined in the statement and checklist of PRISMA (Preferred Reporting Items for Systematic Reviews and Meta-Analyses) [41]. The protocol for this systematic review was registered on INPLASY, and the PRISMA registration number is INPLASY2024110080. The literature search through the databases was carried out for articles published until November 2024 using the following keywords: (“hippocampus volume” OR “hippocampal volume”) AND (walk OR walking OR “step count” OR “free-living physical activity”) AND NOT (mice OR rat OR rodent OR animal).

### 2.2. Research Framework

The Population, Intervention, Comparison, Outcome (PICO) framework was used to define the article inclusion criteria [42,43]. First, Population (P): human subjects with no further restrictions. Second, Intervention (I): any form of walking activity as an exercise-based walking or as a free-living physical activity form of walking. Third, Comparison (C): no comparison was needed, but this systematic review did not focus on walking compared to other physical activity forms and did not focus on gait-based variations in order to focus on the variability in walking itself rather than what causes its variability at this preliminary stage. Lastly, Outcome (O): hippocampal volume changes.

### 2.3. Screening and Inclusion Criteria

The screening process included only English-language peer-reviewed journal articles, excluding grey literature such as books, book chapters, conference papers, notes, retracted papers, and reviews. Afterwards, duplicates were removed. Articles that met the inclusion and exclusion criteria through their title/abstract were selected for full-text reading. Subsequently, the reference lists and citations of the eligible full-text studies were meticulously reviewed to identify additional relevant articles for consideration.

### 2.4. Risk of Bias Assessment

The risk of bias assessment was initially set to be conducted using the PEDro scale [44], which allows for the determination of the quality of randomized controlled trials and the potential risk of bias. Some eligible articles were non-randomized studies, which led to additionally using the Risk of Bias in Non-randomized Studies of Interventions (ROBINS-I) tool [45] for fair assessment of all included studies.

### 2.5. Data Analysis and Synthesis

Data were analyzed and synthesized narratively after summarizing key findings, comparative tables, and conclusive illustrations that derive key relationships, guide future research, and identify existing gaps in the literature.

## 3. Results

**Figure 1:**
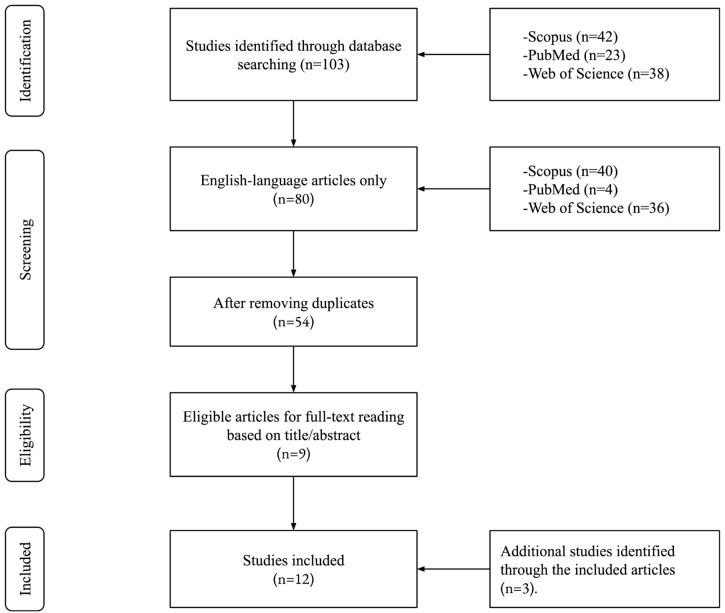
Flow diagram of the study selection process.

**Figure 2:**
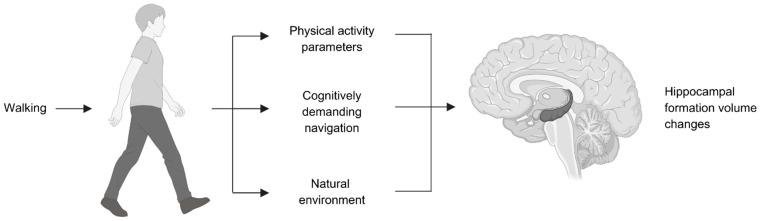
Identified walking factors on hippocampal formation volume increase: physical activity parameters, cognitively demanding navigation, and natural environments.

Despite the fact that only 103 articles were obtained from across the database search (Scopus, PubMed, Web of Science) and the fact that these were reduced to 54 articles after removing duplicates, 9 articles were found eligible for inclusion in this systematic review after reading all titles and abstracts. Forty-five articles were not eligible, as they contained one or more keywords used in the search but their title and abstract were not relevant to the search and inclusion criteria for this systematic review. In addition to the nine eligible studies for inclusion, three more studies were identified while looking through the nine included studies, leading to a total of twelve studies included. The PRISMA flow chart of this study is illustrated in Figure 1, while the walking factors identified through the systematic review are illustrated in Figure 2, before Table 1 presents an overview of all included studies. Table 2 presents the risk of bias scores for the randomized studies using the PEDro scale, while Table 3 presents the risk of bias assessment for the non-randomized studies using the ROBINS-I tool. Both tools showed acceptable scores for all included studies. Afterwards, Table 4 synthesizes the correlative hippocampal formation volume changes dependent on effective parameters (physical activity parameters, navigation training, or physical environment). Table 5 explores the homogeneity and variance in those parameters across all included studies, showing the heterogeneity of reported walking intensity classifications, while the step count categorizations varied. It is also evident that some studies combined moderate and vigorous intensities, while others showed a greater effect only of the latter, which was marginal in some combined cases.

Overall, the included studies link the adaptive hippocampal formation volume changes to factors associated with walking such as the environment [46], environment and walking as physical activity [54], walking physical activity parameters with a diversity of measurements such as intensity, duration, step count, and activity count [47,48,49,50,51,52,55,56], navigational cognitive demands [57], and walking as a 10-year habit [53]. Most studies relied on objective measurements of walking through accelerometers or activity trackers [48,49,50,51,52,55,56], while a few utilized subjective self-reports [47,53]. The scopes are diverse, which urges a cautious interpretation of any synthesis provided in this systematic review.

**Figure 3:**
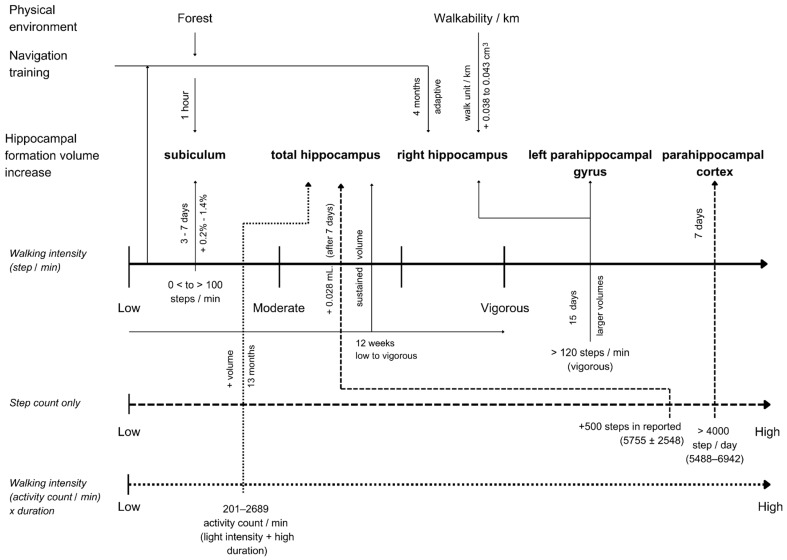
Synthesis of walking and hippocampal formation volume change relationships.

Multiple studies highlighted the benefits of higher intensity and greater amounts of walking on total hippocampal volume and specific regions [48,49,50,51,52,56], while other hippocampal regions, specifically the subiculum, benefit from low-intensity walking and exposure to nature [46,55]. Maintaining walking over time also emerged as important to reducing the atrophy rate in general [53]. Environments with increased walkability and cognitively demanding navigation can benefit the right hippocampus specifically [54,57]. Figure 3 provides an integrative synthesis of all key findings to guide future research and identify the existing gaps in the literature.

Both the PEDro scale and the ROBINS-I tool assessments revealed that most of the included studies achieved generally acceptable scores, reflecting a moderate level of methodological quality and a moderate risk of bias. These results suggest that despite some areas needing enhancement—particularly in terms of subjective measures of walking [47,53], subjective measures of walking in objectively defined residential buffer diameters [54], and unequal combination of moderate and vigorous intensities [49] in some non-randomized studies—the included studies largely adhered to standards that should yield dependable and credible findings.

## 4. Discussion

Though very few studies were found to explain the impact of walking on the hippocampal formation volume change (*n* = 12), all studies showed a positive adaptive effect despite the heterogeneity of scopes (environment, navigation, and physical activity) and measurements used to define the physical activity parameters. Studies focusing on specific hippocampal formation volume changes explained the subregional sensitivity to specific walking factors compared to studies reporting the effect on the total hippocampal volume, and studies using objective measures of walking provided more insights that were not captured using self-reported measures of physical activity in some cases.

Some studies relied on objective measurements of physical activity [48,49,50,51,52,55,56], while others relied on self-reports about physical activity [47,53,54]. Several studies tested the change in total hippocampal volume and subregions, but no studies tested the change in the dentate gyrus volume, which recalls the earlier discussion that adult hippocampal neurogenesis is debatable and that any increase in volume can represent other plasticities and not neurogenesis, such as synaptic remodeling and dendritic branching.

Only one study explored the mediating effect of BDNF in the observed change in hippocampus volume [51]. This supports our earlier emphasis on not overgeneralizing the positive correlation between physical activity and each BDNF and hippocampus volume change, as it appears that it is a more complex interrelationship that requires further research, since BDNF is often consumed for brain uptake or muscle repair as part of the complex BDNF regulation process [58].

### 4.1. Total Hippocampal Volume Changes Through Walking and Parameters

**Figure 4:**
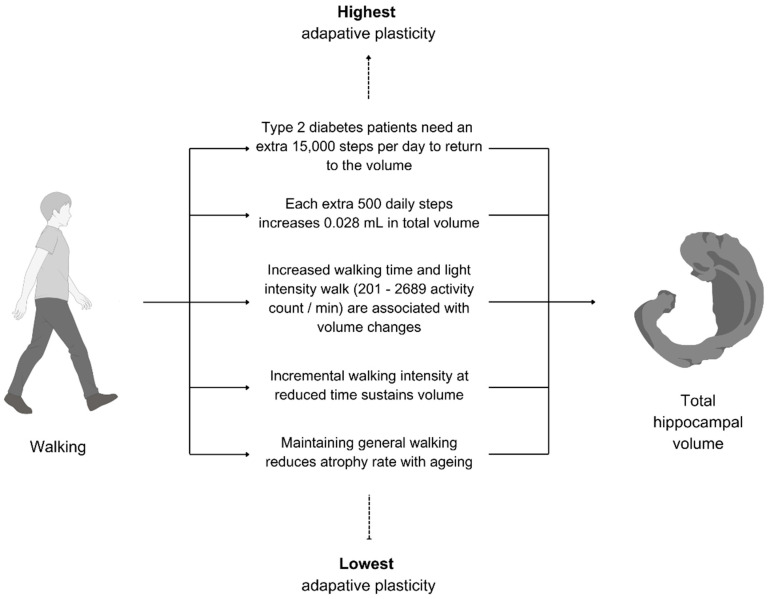
Walking parameters associated with total hippocampal volume changes.

Despite the scarcity of studies, the common focus on total hippocampal volume allowed for more insights into what walking factors, specifically those related to physical activity, affect the total hippocampal volume. As shown in Figure 4, walking was found to be effective for maintaining, increasing, and even reversing the hippocampal atrophy associated with certain health conditions (e.g., type 2 diabetes).

Healthy individuals can highly benefit from these insights, and also, since type 2 diabetes patients can benefit from a customized step count range, subjects with mental health disorders, neurological conditions, and metabolic and cardiovascular conditions, where subjects have reduced hippocampal volume compared to normal subjects, can strongly build on the findings in this systematic review. First, future mental-health-based research can build on these insights in order to experiment with increasing the hippocampal volume through walking in mental health conditions such as borderline personality disorder (BPD) [59], post-traumatic stress disorder (PTSD) [60], clinical depression [61], and bipolar disorder and schizophrenia [62]. Where aerobic exercise is proposed as a tool to improve hippocampal plasticity in humans for mental health [63], we strongly urge focusing on walking as a habit-based, cost-free physical activity. Second, hippocampal atrophy is a hallmark of Alzheimer’s disease (AD) and mild cognitive impairment (MCI) [64,65], as well as of multiple sclerosis, where the latter was proven to show preserved hippocampal volume through walking, as proven in this systematic review in the study by Sandroff et al. [50]. We strongly recommend that walking may achieve cognitive preservation for patients with AD and MCI, urging future research to experiment based on the insights from this systematic review, since physical exercise has been explored in relation to both AD and MCI recently [66,67]. Last but not least, a number of metabolic and cardiovascular conditions such as obesity and diabetes can show hippocampal volume variations, where type 2 diabetes was explored by Zabetian-Targhi et al. [49], providing useful insights for the future consideration of step counts towards returning to a good hippocampal volume in the case of obesity [68].

The challenge will be consistently fulfilling walking physical activity or step count parameters. One way to achieve this is by relying on treadmill use. However, for higher consistency, we urge further exploration and experimentation into the recent hypothesis about environmental affordance for physical activity, aiming to promote the means through which the built environment (urban or architectural) can be designed to increase the potential of reaching sufficient metabolic equivalents (METs) [35]. Physical activity quantified about METs is found to be associated with hippocampal volume [69], urging further research into the environmental role in increasing walking and step counts for brain health.

### 4.2. Right-Hippocampal Volume Changes Through Walking in Built Environments

**Figure 5:**
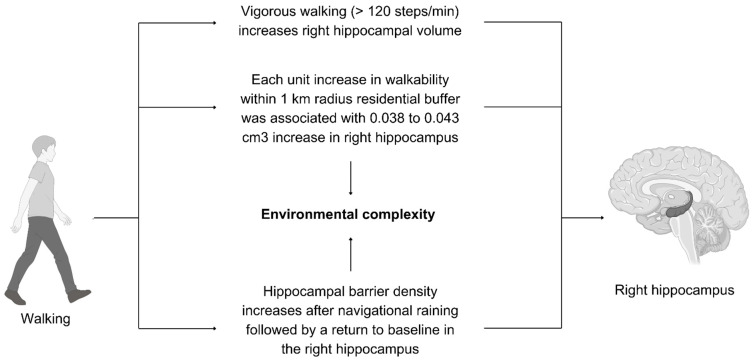
Environmental walking parameters associated with the right hippocampus.

While in the previous section we encouraged designing environments to increase walking for a potential increase in total hippocampal volume, we also emphasized that specific built environment parameters can specifically increase the right hippocampus, as evidenced by Cerin et al. [54] and Lövdén et al. [57] in this systematic review, as shown in Figure 5, while vigorous walking was specifically also reported to be positively associated with the right hippocampus volume [48].

**Figure 6:**
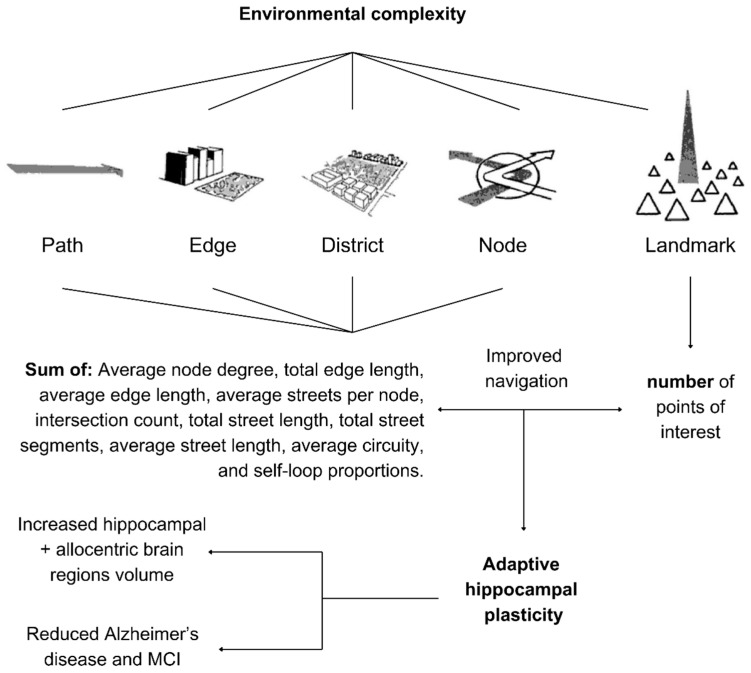
Environmental complexity as explained by Shin et al. [70] and Yuan and Kennedy [71].

The right hippocampus is not only adaptive to walking as a physical activity but also, most importantly, spatial variables. Future research is encouraged to explore environmental complexity to increase walkability as well as navigational learning, as shown in Figure 6, as per the studies by Shin et al. [70] and Yuan and Kennedy [71], and it has also proven to be effective among rodents [72]. This approach is auspicious for pedestrians, since early studies on London taxi drivers showed that cognitively demanding spatial navigation positively affected hippocampal structural changes. Right-hippocampal volume was correlated with the amount of time spent as a taxi driver (positively in the posterior and negatively in the anterior hippocampus) [73], where London taxi drivers even had a better structural adaptability than bus drivers [74]. Similar findings were found regarding ambulance drivers, similar to taxi drivers, regarding navigation and spatial processing [75], which urges implementing environmental complexity for pedestrians, as shown in Figure 6.

Environmental complexity was quantified in a few studies. Yuan and Kennedy [71] developed their construct of environmental complexity after Kevin Lynch’s five elements in his *The Image of the City* book: paths, edges, districts, nodes, and landmarks, emphasizing that there is a necessity to step beyond relying on single elements and emphasizing that their patterning create the needed complexity [76,77]. They obtained data for paths, edges, and nodes from measures of street networks, while points of interest (POIs) captured landmarks with diverse geographic characteristics, physical structures, buildings, shops, malls, educational institutions and schools, industrial buildings, government facilities, and others. The quantification of networks was developed after Boeing’s network measures were implemented to quantify the geometric, topologic, and positional characteristics of road networks [78]. Yuan and Kennedy [71] calculated thirteen network measures related to the city and selected the four least-correlated network measures (which we see as in line with the concept of synchronization of increasing quantity with diversity for spatial complexity), resulting in focusing on intersection count, average streets per node, average street length, and average circuitry. This approach was argued to be well reflective of Lynch’s image of the city theory. Shin et al. [70] employed ten street-network metrics and POIs, totaling 20 measures to assess environmental complexity. Each zip-code zone was scored from 0 to 20 based on these metrics. Thus, the right hippocampus is adaptive to vigorous-intensity walking, increased walkability, and navigational complexity in the urban environment. If the design of urban environments can achieve these three parameters, then it could be ideal for the right hippocampus rather than relying on a long route compared to more complex navigation.

### 4.3. Left-Hippocampal Changes: Current Gaps

In their study, Cerin et al. [54] found no significant relationship between neighborhood walkability and the left hippocampus, and the focus on this specific hippocampal region was significantly missing among the few studies available. Cerin et al. [54] explained that the hippocampal volume change was region-specific due to spatial information retention and location memory. While the impact of walking on left-hippocampal volume was not explored in the studies included in this systematic review, an earlier systematic review and meta-analysis showed that aerobic exercise had significant positive effects on the left-hippocampal volume [31], which urges future research to explore how walking can also be effective for left-hippocampal changes.

### 4.4. Subiculum Volume Change Through Low-Intensity Walking and Natural Environment

Besides its role in spatial orientation and memory [23], the subiculum plays a role in the inhibition of the hypothalamic–pituitary–adrenal (HPA) axis’s stress-associated response [79]. Sudimac and Kühn [46] explained that exposure to nature had a beneficial effect on the subiculum, since it is associated with stress response inhibition and anxiety control, which is similar to how nature decreases stress-related amygdala activity [80]. However, they also explained that it is possible that the increase in subiculum volume may be associated with the task of navigating through a novel environment. They further explained and supported our initial argument about neurogenesis, in that synaptogenesis, glial processes, and dendritic branching occur on a short-term scale, unlike neurogenesis, despite the debate.

Still, it is unclear what natural features (e.g., color, olfactory enrichment, natural sounds, etc.) influence the observed positive changes in the subiculum in response to nature [46]. The increased subiculum volume is also hypothesized to be due to abundant oxygen in forests in comparison with urban spaces. Such ambiguity, however, provides opportunities for future research on biophilic design to experiment with different biophilia scores [81] or biophilic designs [82,83]. Since subiculum plasticity occurs on a short-term scale, future research could feasibly explore the biophilic scores or factors that can promote adaptive subiculum plasticity and also potentially reduce amygdala activity.

Sudimac and Kühn [46] also hypothesized that the increase in subiculum volume could be due to exposure to novelty in the forest environment, which is a hypothesis we support in this systematic review through key rodent-based studies and reviews that show a reduction in anxiety through environmental novelty [72,84,85].

**Figure 7:**
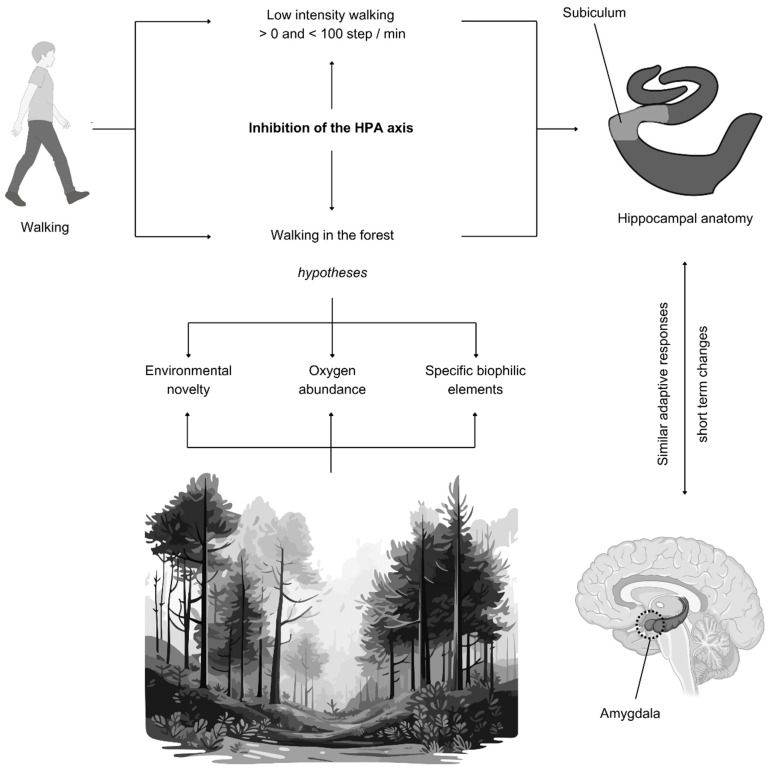
Subiculum volume increases in response to low-intensity walking and nature.

Figure 7 maps out the complex walking–environment–subiculum dynamics to guide future research. We urge future research to experiment on subjects proven to benefit from HPA axis stress responses at this preliminary stage of exploring the elements in nature or novelty responsible for subiculum plasticity.

Those subiculum–amygdala changes may be gender-sensitive, and they may also be more beneficial to subjects with higher emotional reactivity, such as BPD patients. On the one hand, Varma et al. [55] showed that walking with a low intensity is associated with the subiculum in women. Interestingly, an amygdala activity decrease was also found to occur in women in another study [86], which urges careful consideration in future research. On the other hand, Khalil [59] showed that BPD patients have greater amygdala reactivity.

The long-term effects of walking in natural environments on the subiculum are worth exploring, since the subiculum responds similarly to the amygdala to stress and natural environments, where the amygdala has been shown to benefit from years-long exposure to forests [87].

### 4.5. Parahippocampal Gyrus Volume Change Through Vigorous Walking and High Step Count

The parahippocampal gyrus is anatomically located adjacent to the hippocampus and comprises multiple subregions, including the entorhinal cortex and parahippocampal cortex [88]. The region plays an important role in memory encoding and retrieval. For instance, atrophy in the parahippocampal gyrus has been observed in Alzheimer’s disease and is associated with early memory impairments [89].

**Figure 8:**
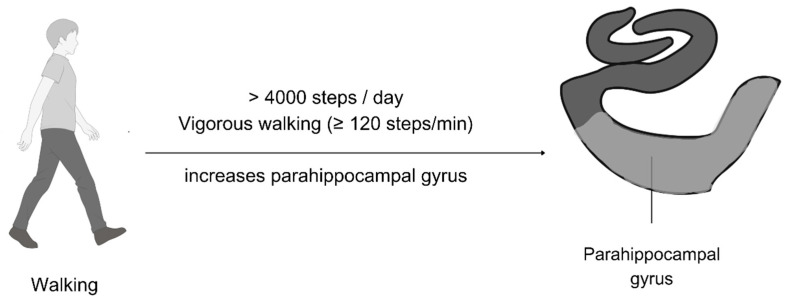
Parahippocampal gyrus volume increases in response to high-intensity walking.

Two studies in this systematic review showed that a higher step count (>4000 steps/day) was associated with an increase in the parahippocampal cortex [52] and that vigorous walking (≥120 steps/min) was associated with a larger left-parahippocampal gyrus [48]. Both studies suggest that higher-level physical activity is better for the parahippocampal gyrus, unlike earlier studies showing that lower-level physical activity is better for the subiculum. Figure 8 illustrates the effective walking parameters needed to increase the parahippocampal gyrus volume, which can be facilitated using treadmills or through the environment [35].

### 4.6. Dentate Gyrus Changes: The Ongoing Gap

With the debate on adult hippocampal neurogenesis still ongoing, this systematic review fails to add to the existing debate, since none of the included studies reported results on the dentate gyrus through walking. At this point, it is unclear whether walking can at least sustain the dentate gyrus volume even if the increase in adult hippocampal neurogenesis is debatable, and it is unclear what physical activity parameters may be sufficient in that regard. This area of research is critically important, since recent research aims to explore the ways that environmental affordance for walking and climbing stairs can promote neurosustainability [35].

### 4.7. Limitations, Future Research, and Practical Implications

Practical implications include implementing walking prescriptions adapted to needs, emphasizing navigation complexity, leveraging biophilic design, and creating walkable built environments. However, this systematic review has some limitations that provide avenues for future research. First, the small number of eligible studies constrains any generalizability of the synthesis. More research is needed, especially employing consistent objective measures of walking parameters and hippocampal subregions. Second, the left hippocampus is under-studied; its association with walking requires elucidation. Third, no studies examined the dentate gyrus, warranting investigation in light of the neurogenesis debate. Fourth, mediators like BDNF need further analysis. Fifth, little research focuses on subregional responses to walking, which limits a broader understanding of the hippocampal formation volume changes. Last but not least, mediating walking through the built environment is a promising research area that remains unexplored [35]. At this point, treadmills can be used to conduct more experiments, but it is evident through this systematic review that the impact of navigation and environmental qualities are inseparable, which proposes relying more on real-world environments.

## 5. Conclusions

This systematic review provides preliminary evidence that walking can positively affect hippocampal volume, counteracting age-related atrophy. While limited studies were available, the hippocampus seems sensitive to modifiable factors like walking intensity, amount, environment, and navigation demands. Elucidating subregion-specific effects is critical for optimizing interventions. Key future directions include methodologically rigorous research on the under-studied left hippocampus and dentate gyrus, long-term interventions, consistent objective measures, mediators like BDNF, comparisons with other activities, clinical populations, and practical applications leveraging environmental design and walking prescriptions. Insights into hippocampal plasticity mechanisms can be translated into lifestyle recommendations and built environments, promoting cognitive health through this accessible activity. Overall, this systematic review highlights the promising neuroprotective potential of walking that is worthy of further investigation.

## Figures and Tables

**Table 1:** Overview of included studies.

Author/s and Publication Year	Walking Factor	Sample	Relevant Aim/s and Measurements	Results	Author/s’ Remarks on Potential Effects
Sudimac and Kühn [46]	Environment (forest vs. urban)	60 participants (27.31 ± 6.74 years old).	Explore the impact of a 1 h walk in nature (forest) versus an urban environment (street) on hippocampal formation.	Walking in nature increased the subiculum volume.	The observed effect could potentially be influenced by factors such as the higher oxygen levels or to specific natural variables (green color, odours, sounds, etc.).
Rodriguez-Ayllon et al. [47]	Physical activity	3027 participants (62.45 ± 7.27 years old).	Explore the bidirectional relationship between leisure-time physical activity (self-reported duration, frequency, and type of leisure activity) and brain structures (2-year gap).	Walking (2.45 ± 3.05 h per week) at baseline and (2.58 ± 3.25 h per week) at follow-up was not significantly associated with hippocampal volume.	Walking enrichment (e.g., green vs. urban spaces, different vs. same route, solitary vs. group walk) and walking speed.
Domingos et al. [48]	Physical activity	110 participants (68.42 ± 3.12 years old).	Explore the association between physical activity (objectively measured using the Xiaomi Mi Band 2^®^ for 15 consecutive days) and brain function and structure.	Higher time spent in vigorous walking (≥120 steps/min) was associated with larger left parahippocampal gyrus and right hippocampus volumes.	There was a greater functional connectivity (FC) between the frontal gyrus, cingulate gyrus, and occipital inferior lobe for physical activity. Sedentary time had a relationship with lower FC in the exact networks.
Zabetian-Targhi et al. [49]	Physical activity	165 participants (68.3 ± 6.6 years old).	Determine the association between physical activity intensity and step count (using an accelerometer to quantify step count and physical activity intensity over 7 days) with brain structure.	Higher step count had a significant relationship with greater hippocampal volume (β = 0.028, 95% CI = 0.005, 0.051).	The association between each extra 500 daily steps and 0.028 mL of the hippocampal volume was consistent with the previous literature, adding that individuals with type 2 diabetes (T2D) with lower hippocampal volume needed an additional 15,000 steps per day.
Sandroff et al. [50]	Physical activity	11 participants in a pilot study (46.3 ± 11 years old).	Investigate the impact of treadmill walking, starting with 15–20 min at light-to-moderate intensity and gradually increasing to 40 min at vigorous intensity by the 12th week on the hippocampus.	A 12-week treadmill walking program (3 days per week) was linked to the maintenance of normalized hippocampal volume in people with multiple sclerosis (MS).	The intervention condition resulted in significant reductions in connectivity between the hippocampus and other regions of the DMN, representing a beneficial adaptation.
Bergman et al. [51]	Physical activity	80 participants (40–67 years old).	Examine the long-term impact on brain function following the installation of treadmill workstations in offices over a 13-month period using activPAL3 devices.	High walking time and light-intensity walking physical activity (201–2689 counts per minute) associated with hippocampal volume changes not mediated by changes in BDNF.	The impact on BDNF may vary depending on the intensity level of physical activity, warranting further investigation.
Siddarth et al. [52]	Physical activity	26 participants (72.7 ± 8.1 years old).	Examine the association between physical activity (for 7 days by accelerometers) with hippocampal and neighboring cortical sub-regions.	Higher activity (>4000 steps/day) associated with thicker parahippocampal cortex (median difference = 0.12 m, ES = 0.93, *p* = 0.04).	A higher step count was linked to improved performance in attention, information-processing speed, and executive functioning but not with memory recall.
Best et al. [53]	Physical activity	141 participants (70–79 years old).	Examine the effects of maintaining physical activity (self-reported walking time) over 13 years on brain structure.	A 1 SD increase in the walking slope score showed a 4.5% decrease in hippocampal volume compared to a 6.2% decrease on average.	Physical activity levels throughout ageing can be an effective contributor to neural and cognitive protection.
Cerin et al. [54]	Physical activity	Integrated samples with a mean age of 75 years.	Assess neighborhood walkability (the International Physical Activity Questionnaire— Long Form (IPAQ-LF)) with brain volume.	Each increase in walkability (unit/1 km radius) was associated with a 0.038 to 0.043 cm^3^ increase in the right hippocampus.	The multifaceted aspects of physical activity accounted for the associations with the right hippocampus due to spatial information retention and location memory [2].
Varma et al. [55]	Physical activity	132 participants (60 years and older).	Explore hippocampal sub-regional specificity of physical activity by measuring walking through an accelerometer.	Daily walking was linked to a larger subiculum surface area in women, even after controlling for self-reported exercise.	Physical activity below the threshold of moderate-intensity exercise may be linked to specific hippocampal subregions not commonly associated with exercise.
Varma et al. [56]	Physical activity	92 participants (67.3 ± 6.1 years old).	Examine whether higher levels of objectively measured daily walking activity, tracked using a step activity monitor, are associated with increased hippocampal volume.	Higher amounts, durations, and frequencies of daily walking each correlated with larger hippocampal volumes, with effect sizes of 0.2–1.4%, compared to annual atrophy rates of 0.8–2.0% in healthy elders.	Modest increases in low-intensity lifestyle activities may support memory and reduce dementia risk.
Lövdén et al. [57]	Navigation (cognitively demanding walking)	56 (20–30 years old), and 62 (60–70 years old).	Explore the effect of treadmill walking at a modest and not physically demanding pace with navigation training using VR compared to walking only.	Hippocampal barrier density increased after training and returned to baseline in the right hippocampus but declined in controls and the left hippocampus.	Low demands of the slow walking speed used in this study are unlikely to cause any major effects on physical fitness. Spatial demands help protect hippocampal integrity from age-related decline.

**Table 2:** Risk of bias scores for randomized controlled studies using the PEDro scale.

Study	PEDro Scale Items										
	1	2	3	4	5	6	7	8	9	10	Total Score
Sudimac and Kühn [46]	Y	N	Y	N	N	N	Y	Y	Y	Y	6
Sandroff et al. [50]	Y	N	Y	N	N	Y	Y	Y	Y	Y	7
Bergman et al. [51]	Y	N	Y	N	N	N	Y	Y	Y	Y	6
Lövdén et al. [57]	Y	N	Y	N	N	Y	Y	Y	Y	Y	7

PEDro scale Items: 1 = random allocation, 2 = concealed allocation, 3 = groups similar at baseline, 4 = participant blinding, 5 = therapist blinding, 6 = assessment blinding, 7 = <15% dropout rate, 8 = intention-to-treat analysis, 9 = between-group differences reported, 10 = point estimate and variability reported. Y = yes. N = no. According to the scale, scores 0–3 are ‘poor’, 4–5 are ‘fair’, 6–8 are ‘good’, and 9–10 are ‘excellent’.

**Table 3:** Risk of bias scores for other non-randomized studies using the ROBINS-I tool.

Study	ROBINS-I Tool							
	D1	D2	D3	D4	D5	D6	D7	Overall
Rodriguez-Ayllon et al. [47]	+	?	x	+	?	+	+	x
Domingos et al. [48]	+	?	+	+	?	+	+	?
Zabetian-Targhi et al. [49]	+	?	+	+	?	x	+	x
Siddarth et al. [52]	+	?	+	+	?	+	+	?
Best et al. [53]	+	?	x	+	?	+	+	x
Cerin et al. [54]	+	?	?	+	?	+	+	?
Varma et al. [55]	+	?	+	+	?	+	+	?
Varma et al. [56]	+	?	+	+	?	+	+	?

Domains: D1 = bias due to confounding, D2 = bias due to selection of participants. D3 = bias in classification of interventions, D4 = bias due to deviations from intended interventions, D5 = bias due to missing data, D6 = bias in measurement of outcomes, D7 = bias in selection of the reported result. Assessment is as follows: + = low risk of bias, ? = moderate risk of bias, x = serious risk of bias.

**Table 4:** Synthesis of reported adaptive hippocampal formation volume changes in relation to specific physical activity parameters, cognitively demanding navigation, and the physical environment.

Author/s and Publication Year	Participants’ Age in Years	Walking Parameters	Effective Parameter	Sig.	Hippocampal Formation Volume Change
Sudimac and Kühn [46]	27.31 ± 6.74	1 h walk in two environments	Natural environment	*p* = 0.034	Increased bilateral subiculum volume
Rodriguez-Ayllon et al. [47]	62.45 ± 7.27	Mean 3.05 h/week walk for 2 years	-	*p* = 0.502	-
Domingos et al. [48]	68.42 ± 3.12	15 days walking at various intensities	Vigorous (≥120 steps/min)	*t* = 3.42 and *t* = 4.43	Larger left-parahippocampal gyrus and right-hippocampal volumes
Zabetian-Targhi et al. [49]	68.3 ± 6.6	Step count and MVPA for 7 days	Increased step count	*p* = 0.019	Greater hippocampal volume
Sandroff et al. [50]	46.3 ± 11	From low to vigorous, 3 days/week, for 12 weeks	Increased intensity	*p* = 0.05	Sustained hippocampal volume
Bergman et al. [51]	40–67	Light-intensity walking for 13 months	Increased time of light walking (201–2689 activity count/min), not intense	*p* = 0.027 (changes in walking time)	Increased hippocampal volume
Siddarth et al. [52]	72.7 ± 8.1	Step count tracked for 7 days	>4000 steps/day	*p* = 0.04	Thicker parahippocampal cortex
Best et al. [53]	70–79	Maintain walking for 13 years	Maintain walking time	*p* = 0.03	Lower reduction in hippocampal volume
Cerin et al. [54]	Mean = 75	Neighborhood walkability	Increase in walkability/1 km home buffer	*p* < 0.05	Increased right-hippocampal volume
Varma et al. [55]	≥60	Tracked daily walking for 3–7 days	Low intensity (>0 steps/min and <100 steps/min)	*p* < 0.05	Larger surface area of the subiculum
Varma et al. [56]	67.3 ± 6.1	Tracked daily walking for 3–7 days	Greater amount, duration, and frequency	*p* < 0.01	Larger hippocampal volume
Lövdén et al. [57]	20–30 and 60–70	Low-intensity walking and navigation	Navigational training	*p* < 0.05	Adaptive right-hippocampal volume

**Table 5:** Homogeneity and variance in walking physical activity measures and parameters.

Author/s and Publication Year	Walking Factor	Walking Measure	Walking Parameters (Duration, Intensity, Step Count, or Activity Count)
Sudimac and Kühn [46]	Environment	-	-
Rodriguez-Ayllon et al. [47]	Physical activity	Time	Results (hour/week) = 2.58 ± 3.25
Domingos et al. [48]	Physical activity	Light, moderate, and vigorous intensities	Steps/min: -Sedentary = 0–19 -Light = 60–99 -Moderate = 100–119 -Vigorous = ≥120 Steps/day: -Light = 3180.82 ± 4651.32 -Moderate = 1883. 34 ± 1988.94 -Vigorous = 1263.68 ± 2311.07 min/day: -Light = 40.06 ± 57.54 -Moderate = 17.24 ± 18.32 -Vigorous = 9.31 ± 16.56
Zabetian-Targhi et al. [49]	Physical activity	Step count and moderate-to-vigorous intensity (combined)	Steps/day: 5755 ± 2548 min/day: 21.8 ± 21.7 (mostly moderate, whereas vigorous = 0.13 ± 0.6)
Sandroff et al. [50]	Physical activity	Incremental intensity	min/day: -Light-to-moderate = 15–20 -Vigorous = 40
Bergman et al. [51]	Physical activity	Time and light and moderate-to-vigorous intensities	Activity count/min: -Light = 201–2689 -Moderate-to-vigorous = ≥2690
Siddarth et al. [52]	Physical activity	Step count	Steps/day: -Lower physical activity = ≤4000 -Higher physical activity = >4000 (5488–6942)
Best et al. [53]	Physical activity	Time	-
Cerin et al. [54]	Physical activity	Spatial walkability	-
Varma et al. [55]	Physical activity	Low and moderate-to-vigorous intensities	Steps/min: -Low = >0 and <100 -Moderate-to-vigorous = ≥100
Varma et al. [56]	Physical activity	Low and moderate-to-vigorous intensities	Steps/min: -Low = >0 and <100 -Moderate-to-vigorous = ≥100
Lövdén et al. [57]	Navigation	-	-

## References

[1] Chauhan P., Jethwa K., Rathawa A., Chauhan G., Mehra S. (2021). The anatomy of the hippocampus. Exon Publ..

[2] Burgess N., Maguire E.A., O’Keefe J. (2002). The human hippocampus and spatial and episodic memory. Neuron.

[3] Pereira A.C., Huddleston D.E., Brickman A.M., Sosunov A.A., Hen R., McKhann G.M., Sloan R., Gage F.H., Brown T.R., Small S.A. (2007). An in vivo correlate of exercise-induced neurogenesis in the adult dentate gyrus. Proc. Natl. Acad. Sci. USA.

[4] Bonfanti L., La Rosa C., Ghibaudi M., Sherwood C.C. (2024). Adult neurogenesis and “immature” neurons in mammals: An evolutionary trade-off in plasticity?. Brain Struct. Funct..

[5] Altman J., Das G.D. (1965). Autoradiographic and histological evidence of postnatal hippocampal neurogenesis in rats. J. Comp. Neurol..

[6] Gage F.H. (2000). Mammalian neural stem cells. Science.

[7] Clemenson G.D., Deng W., Gage F.H. (2015). Environmental enrichment and neurogenesis: From mice to humans. Curr. Opin. Behav. Sci..

[8] Jessberger S., Gage F.H. (2014). Adult neurogenesis: Bridging the gap between mice and humans. Trends Cell Biol..

[9] Eriksson P.S., Perfilieva E., Björk-Eriksson T., Alborn A.M., Nordborg C., Peterson D.A., Gage F.H. (1998). Neurogenesis in the adult human hippocampus. Nat. Med..

[10] Boldrini M., Fulmore C.A., Tartt A.N., Simeon L.R., Pavlova I., Poposka V., Mann J.J., Rosoklija G.B., Stankov A., Arango V. (2018). Human hippocampal neurogenesis persists throughout aging. Cell Stem Cell.

[11] Moreno-Jiménez E.P., Flor-García M., Terreros-Roncal J., Rábano A., Cafini F., Pallas-Bazarra N., Ávila J., Llorens-Martín M. (2019). Adult hippocampal neurogenesis is abundant in neurologically healthy subjects and drops sharply in patients with Alzheimer’s disease. Nat. Med..

[12] Moreno-Jiménez E.P., Terreros-Roncal J., Flor-García M., Rábano A., Llorens-Martín M. (2021). Evidences for adult hippocampal neurogenesis in humans. J. Neurosci..

[13] Spalding K.L., Bergmann O., Alkass K., Bernard S., Salehpour M., Huttner H.B., Frisén J., Boström E., Westerlund I., Vial C. (2013). Dynamics of hippocampal neurogenesis in adult humans. Cell.

[14] Tobin M.K., Musaraca K., Disouky A., Shetti A., Bheri A., Honer W.G., Lazarov O., Kim N., Dawe R.J., Bennett D.A. (2019). Human hippocampal neurogenesis persists in aged adults and Alzheimer’s disease patients. Cell Stem Cell.

[15] Cipriani S., Ferrer I., Aronica E., Kovacs G.G., Verney C., Nardelli J., Adle-Biassette H., Khung S., Delezoide A.-L., Milenkovic I. (2018). Hippocampal radial glial subtypes and their neurogenic potential in human fetuses and healthy and Alzheimer’s disease adults. Cereb. Cortex.

[16] Sorrells S.F., Paredes M.F., Cebrian-Silla A., Sandoval K., Qi D., Kelley K.W., Alvarez-Buylla A., James D., Mayer S., Chang J. (2018). Human hippocampal neurogenesis drops sharply in children to undetectable levels in adults. Nature.

[17] Duque A., Spector R. (2019). A balanced evaluation of the evidence for adult neurogenesis in humans: Implication for neuropsychiatric disorders. Brain Struct. Funct..

[18] Snyder J.S. (2019). Recalibrating the relevance of adult neurogenesis. Trends Neurosci..

[19] Simard S., Matosin N., Mechawar N. (2024). Adult Hippocampal Neurogenesis in the Human Brain: Updates, Challenges, and Perspectives. Neuroscientist.

[20] Cherednichenko A., Miró-Padilla A., Adrián-Ventura J., Monzonís-Carda I., Beltran-Valls M.R., Moliner-Urdiales D., Ávila C. (2024). Physical activity and hippocampal volume in young adults. Brain Imaging Behav..

[21] O’Keefe J., Nadel L. (1978). The Hippocampus as a Cognitive Map.

[22] Keresztes A., Raffington L., Bender A.R., Bögl K., Heim C., Shing Y.L. (2022). Longitudinal developmental trajectories do not follow cross-sectional age associations in hippocampal subfield and memory development. Dev. Cogn. Neurosci..

[23] O’Mara S. (2005). The subiculum: What it does, what it might do, and what neuroanatomy has yet to tell us. J. Anat..

[24] De Nys L., Anderson K., Ofosu E.F., Ryde G.C., Connelly J., Whittaker A.C. (2022). The effects of physical activity on cortisol and sleep: A systematic review and meta-analysis. Psychoneuroendocrinology.

[25] Goyal M., Singh S., Sibinga E.M., Gould N.F., Rowland-Seymour A., Sharma R., Haythornthwaite J.A. (2014). Meditation programs for psychological stress and well-being: A systematic review and meta-analysis. JAMA Intern. Med..

[26] Loprinzi P.D. (2019). The effects of physical exercise on parahippocampal function. Physiol. Int..

[27] Bartsch T., Wulff P. (2015). The hippocampus in aging and disease: From plasticity to vulnerability. Neuroscience.

[28] Leuner B., Gould E. (2010). Structural plasticity and hippocampal function. Annu. Rev. Psychol..

[29] Thompson D.K., Wood S.J., Doyle L.W., Warfield S.K., Egan G.F., Inder T.E. (2009). MR-determined hippocampal asymmetry in full-term and preterm neonates. Hippocampus.

[30] Feter N., Penny J.C., Freitas M.P., Rombaldi A.J. (2018). Effect of physical exercise on hippocampal volume in adults: Systematic review and meta-analysis. Sci. Sports.

[31] Firth J., Stubbs B., Vancampfort D., Schuch F., Lagopoulos J., Rosenbaum S., Ward P.B. (2018). Effect of aerobic exercise on hippocampal volume in humans: A systematic review and meta-analysis. Neuroimage.

[32] Maasakkers C.M., Weijs R.W., Dekkers C., Gardiner P.A., Ottens R., Rikkert M.G.O., Melis R.J., Thijssen D.H., Claassen J.A. (2022). Sedentary behaviour and brain health in middle-aged and older adults: A systematic review. Neurosci. Biobehav. Rev..

[33] de Oliveira Segundo V.H., de Azevedo K.P.M., de Medeiros G.C.B.S., Mata Á.N.S., Piuvezam G. (2024). Association between sedentary behavior and Brain-Derived Neurotrophic Factor (BDNF) in children and adolescents: A protocol for systematic review and meta-analysis. PLoS ONE.

[34] van der Sluys M.E., Marhe R., van der Laan P.H., Popma A., Scherder E.J.A. (2022). Brief report: Free-living physical activity levels and cognitive control in multi-problem young adults. Front. Hum. Neurosci..

[35] Khalil M.H. (2024). Environmental Affordance for Physical Activity, Neurosustainability, and Brain Health: Quantifying the Built Environment’s Ability to Sustain BDNF Release by Reaching Metabolic Equivalents (METs). Brain Sci..

[36] Barch D.M., Tillman R., Kelly D., Whalen D., Gilbert K., Luby J.L. (2019). Hippocampal volume and depression among young children. Psychiatry Res. Neuroimaging.

[37] Bremner J.D., Narayan M., Anderson E.R., Staib L.H., Miller H.L., Charney D.S. (2000). Hippocampal volume reduction in major depression. Am. J. Psychiatry.

[38] Videbech P., Ravnkilde B. (2004). Hippocampal volume and depression: A meta-analysis of MRI studies. Am. J. Psychiatry.

[39] McCormick B.P., Brusilovskiy E., Snethen G., Klein L., Townley G., Salzer M.S. (2022). Getting out of the house: The relationship of venturing into the community and neurocognition among adults with serious mental illness. Psychiatr. Rehabil. J..

[40] Khalil M.H., Steemers K. (2024). Housing environmental enrichment, lifestyles and public health indicators of neurogenesis in humans: A pilot study. Int. J. Environ. Res. Public Health.

[41] Moher D., Liberati A., Tetzlaff J., Altman D.G., PRISMA Group (2009). Preferred reporting items for systematic reviews and meta-analyses: The PRISMA statement. Ann. Intern. Med..

[42] Schardt C., Adams M.B., Owens T., Keitz S., Fontelo P. (2007). Utilization of the PICO framework to improve searching PubMed for clinical questions. BMC Med. Inform. Decis. Mak..

[43] Farrugia P., Petrisor B.A., Farrokhyar F., Bhandari M. (2010). Research questions, hypotheses and objectives. Can. J. Surg..

[44] De Morton N.A. (2009). The PEDro scale is a valid measure of the methodological quality of clinical trials: A demographic study. Aust. J. Physiother..

[45] Sterne J.A., Hernán M.A., Reeves B.C., Savović J., Berkman N.D., Viswanathan M., Higgins J.P. (2016). ROBINS-I: A tool for assessing risk of bias in non-randomised studies of interventions. BMJ.

[46] Sudimac S., Kühn S. (2024). Can a nature walk change your brain? Investigating hippocampal brain plasticity after one hour in a forest. Environ. Res..

[47] Rodriguez-Ayllon M., Neumann A., Hofman A., Vernooij M.W., Neitzel J. (2024). The bidirectional relationship between brain structure and physical activity: A longitudinal analysis in the UK Biobank. Neurobiol. Aging.

[48] Domingos C., Picó-Pérez M., Magalhães R., Moreira M., Sousa N., Pêgo J.M., Santos N.C. (2021). Free-living physical activity measured with a wearable device is associated with larger hippocampus volume and greater functional connectivity in healthy older adults: An observational, cross-sectional study in northern portugal. Front. Aging Neurosci..

[49] Zabetian-Targhi F., Srikanth V.K., Beare R., Breslin M., Moran C., Wang W., Wu F., Smith K.J., Callisaya M.L. (2021). The association between physical activity intensity, cognition, and brain structure in people with type 2 diabetes. J. Gerontol. Ser. A.

[50] Sandroff B.M., Wylie G.R., Baird J.F., Jones C.D., Diggs M.D., Genova H., Bamman M.M., Cutter G.R., DeLuca J., Motl R.W. (2021). Effects of walking exercise training on learning and memory and hippocampal neuroimaging outcomes in MS: A targeted, pilot randomized controlled trial. Contemp. Clin. Trials.

[51] Bergman F., Matsson-Frost T., Jonasson L., Chorell E., Sörlin A., Wennberg P., Boraxbekk C.J., Öhberg F., Ryberg M., Levine J.A. (2020). Walking time is associated with hippocampal volume in overweight and obese office workers. Front. Hum. Neurosci..

[52] Siddarth P., Rahi B., Emerson N.D., Burggren A.C., Miller K.J., Bookheimer S., Merrill D.A. (2018). Physical activity and hippocampal sub-region structure in older adults with memory complaints. J. Alzheimer’s Dis..

[53] Best J.R., Rosano C., Aizenstein H.J., Tian Q., Boudreau R.M., Ayonayon H.N., Study B.C., Satterfield S., Simonsick E.M., Studenski S. (2017). Long-term changes in time spent walking and subsequent cognitive and structural brain changes in older adults. Neurobiol. Aging.

[54] Cerin E., Rainey-Smith S.R., Ames D., Lautenschlager N.T., Macaulay S.L., Fowler C., Ellis K., Robertson J.S., Rowe C.C., Maruff P. (2017). Associations of neighborhood environment with brain imaging outcomes in the Australian Imaging, Biomarkers and Lifestyle cohort. Alzheimer’s Dement..

[55] Varma V.R., Tang X., Carlson M.C. (2016). Hippocampal sub-regional shape and physical activity in older adults. Hippocampus.

[56] Varma V.R., Chuang Y.F., Harris G.C., Tan E.J., Carlson M.C. (2015). Low-intensity daily walking activity is associated with hippocampal volume in older adults. Hippocampus.

[57] Lövdén M., Schaefer S., Noack H., Bodammer N.C., Kühn S., Heinze H.J., Lindenberger U., Düzel E., Bäckman L. (2012). Spatial navigation training protects the hippocampus against age-related changes during early and late adulthood. Neurobiol. Aging.

[58] Khalil M.H. (2024). The BDNF-Interactive Model for Sustainable Hippocampal Neurogenesis in Humans: Synergistic Effects of Environmentally-Mediated Physical Activity, Cognitive Stimulation, and Mindfulness. Int. J. Mol. Sci..

[59] Khalil M.H. (2025). Borderline in a linear city: Urban living brings borderline personality disorder into crisis through neuroplasticity—An urgent call to action. Front. Psychiatry.

[60] Bremner J.D., Hoffman M., Afzal N., Cheema F.A., Novik O., Ashraf A., Brummer M., Nazeer A., Goldberg J., Vaccarino V. (2021). The environment contributes more than genetics to smaller hippocampal volume in Posttraumatic Stress Disorder (PTSD). J. Psychiatr. Res..

[61] Oyarce D.A.E., Shaw M.E., Alateeq K., Cherbuin N. (2020). Volumetric brain differences in clinical depression in association with anxiety: A systematic review with meta-analysis. J. Psychiatry Neurosci..

[62] Sato J., Hirano Y., Hirakawa N., Takahashi J., Oribe N., Kuga H., Onitsuka T., Nakamura I., Hirano S., Ueno T. (2021). Lower hippocampal volume in patients with schizophrenia and bipolar disorder: A quantitative MRI study. J. Pers. Med..

[63] Kandola A., Hendrikse J., Lucassen P.J., Yücel M. (2016). Aerobic exercise as a tool to improve hippocampal plasticity and function in humans: Practical implications for mental health treatment. Front. Hum. Neurosci..

[64] Park H.Y., Suh C.H., Heo H., Shim W.H., Kim S.J. (2022). Diagnostic performance of hippocampal volumetry in Alzheimer’s disease or mild cognitive impairment: A meta-analysis. Eur. Radiol..

[65] Rao G., Gao H., Wang X., Zhang J., Ye M., Rao L. (2023). MRI measurements of brain hippocampus volume in relation to mild cognitive impairment and Alzheimer disease: A systematic review and meta-analysis. Medicine.

[66] Kress G.T., Popa E.S., Merrill D.A., Bramen J.E., Siddarth P. (2024). The impact of physical exercise on hippocampal atrophy in mild cognitive impairment and Alzheimer’s disease: A meta-analysis. NeuroReport.

[67] Liu S., Yang Y., Wang K., Zhang T., Luo J. (2024). A study on the impact of acute exercise on cognitive function in Alzheimer’s disease or mild cognitive impairment patients: A narrative review. Geriatr. Nurs..

[68] Cherbuin N., Sargent-Cox K., Fraser M., Sachdev P., Anstey K.J. (2015). Being overweight is associated with hippocampal atrophy: The PATH Through Life Study. Int. J. Obes..

[69] Machida M., Takamiya T., Amagasa S., Murayama H., Fujiwara T., Odagiri Y., Shobugawa Y. (2022). Objectively measured intensity-specific physical activity and hippocampal volume among community-dwelling older adults. J. Epidemiol..

[70] Shin N., Rodrigue K.M., Yuan M., Kennedy K.M. (2024). Geospatial environmental complexity, spatial brain volume, and spatial behavior across the Alzheimer’s disease spectrum. Alzheimer’s Dement. Diagn. Assess. Dis. Monit..

[71] Yuan M., Kennedy K.M. (2023). Utility of environmental complexity as a predictor of alzheimer’s disease diagnosis: A big-data machine learning approach. J. Prev. Alzheimer’s Dis..

[72] Khalil M.H. (2024). Environmental enrichment: A systematic review on the effect of a changing spatial complexity on hippocampal neurogenesis and plasticity in rodents, with considerations for translation to urban and built environments for humans. Front. Neurosci..

[73] Maguire E.A., Gadian D.G., Johnsrude I.S., Good C.D., Ashburner J., Frackowiak R.S., Frith C.D. (2000). Navigation-related structural change in the hippocampi of taxi drivers. Proc. Natl. Acad. Sci. USA.

[74] Maguire E.A., Woollett K., Spiers H.J. (2006). London taxi drivers and bus drivers: A structural MRI and neuropsychological analysis. Hippocampus.

[75] Patel V., Liu M., Worsham C.M., Jena A.B. (2024). Alzheimer’s disease mortality among taxi and ambulance drivers: Population based cross sectional study. BMJ.

[76] Banai R. (1999). A methodology for The Image of the City. Environ. Plan. B Plan. Des..

[77] Lynch K. (1960). The image of the environment. Image City.

[78] Boeing G. (2017). OSMnx: New methods for acquiring, constructing, analyzing, and visualizing complex street networks. Comput. Environ. Urban Syst..

[79] Herman J.P., Mueller N.K. (2006). Role of the ventral subiculum in stress integration. Behav. Brain Res..

[80] Sudimac S., Sale V., Kühn S. (2022). How nature nurtures: Amygdala activity decreases as the result of a one-hour walk in nature. Mol. Psychiatry.

[81] Salingaros N.A. (2019). The biophilic healing index predicts effects of the built environment on our wellbeing. JBU—J. Biourbanism.

[82] Huntsman D.D., Bulaj G. (2022). Healthy dwelling: Design of biophilic interior environments fostering self-care practices for people living with migraines, chronic pain, and depression. Int. J. Environ. Res. Public Health.

[83] Zhong W., Schröder T., Bekkering J. (2022). Biophilic design in architecture and its contributions to health, well-being, and sustainability: A critical review. Front. Archit. Res..

[84] Gray J.A. (1982). Précis of The neuropsychology of anxiety: An enquiry into the functions of the septo-hippocampal system. Behav. Brain Sci..

[85] Naber P.A., Witter M.P., Lopes da Silva F.H. (2000). Networks of the Hippocampal Memory System of the Rat: The Pivotal Role of the Subiculum. Ann. N. Y. Acad. Sci..

[86] Sudimac S., Kühn S. (2022). A one-hour walk in nature reduces amygdala activity in women, but not in men. Front. Psychol..

[87] Kühn S., Düzel S., Eibich P., Krekel C., Wüstemann H., Kolbe J., Lindenberger U., Martensson J., Goebel J., Gallinat J. (2017). In search of features that constitute an “enriched environment” in humans: Associations between geographical properties and brain structure. Sci. Rep..

[88] Raslau F.D., Mark I.T., Klein A.P., Ulmer J.L., Mathews V., Mark L.P. (2015). Memory part 2: The role of the medial temporal lobe. Am. J. Neuroradiol..

[89] Zhu L., Wang Z., Du Z., Qi X., Shu H., Liu D., Zhang Z., Su F., Ye Q., Liu X. (2020). Impaired Parahippocampal Gyrus–Orbitofrontal Cortex Circuit Associated with Visuospatial Memory Deficit as a Potential Biomarker and Interventional Approach for Alzheimer Disease. Neurosci. Bull..

